# *Candida Oleophila* Proliferated and Accelerated Accumulation of Suberin Poly Phenolic and Lignin at Wound Sites of Potato Tubers

**DOI:** 10.3390/foods10061286

**Published:** 2021-06-04

**Authors:** Xiaoyuan Zheng, Hong Jiang, Esrat Mahmud Silvy, Shijia Zhao, Xiuwei Chai, Bin Wang, Zhicheng Li, Yang Bi, Dov Prusky

**Affiliations:** 1 College of Food Science and Engineering, Gansu Agricultural University, Lanzhou 730070, China; xiaoyuanzheng1@163.com (X.Z.); 13893604069@163.com (H.J.); silvy.sonia@yahoo.com (E.M.S.); zhaoshijia_123@163.com (S.Z.); 18794852382@163.com (X.C.); wangbin_1519@163.com (B.W.); gsau_lzc11@163.com (Z.L.); dovprusk@volcani.agri.gov.il (D.P.); 2 Department of Postharvest Science of Fresh Produce, Agricultural Research Organization, Rishon LeZion 7505101, Israel

**Keywords:** *Candida oleophila*, postharvest tuber, wound healing, suberin poly phenolic, lignin, phenylpropanoid metabolism

## Abstract

*Candida oleophila* is a type of biocontrol yeast offering effective postharvest disease control. To the best of our knowledge, the effect of *C. oleophila* upon the healing of tubers is yet to be studied. The present study addresses the existing knowledge gap by investigating the effect of *C. oleophila* on wound healing in potato tubers. The results show that *C. oleophila* colonized and proliferated at the wound sites during the early and intermediate stages of healing. In addition, *C. oleophila* reduced weight loss of wounded tubers, decreased disease index of inoculated tubers with *Fusarium sulphureum*, and accelerated accumulation of suberin poly phenolic (SPP) and lignin at wound sites. *C. oleophila* activated phenylpropanoid metabolism and increased the content of SPP monomers, total phenol, flavonoids, and lignin. Furthermore, the yeast increased H_2_O_2_ content as well as peroxidase activity.

## 1. Introduction

Wound healing is a typical biological characteristic in postharvest potato tubers [1,2]. Suberin poly phenolic (SPP) and lignin are critical components of wounded tissue, providing primary protection against infection by pathogens and evaporation [3]. However, natural wound healing takes a long time, leading to considerable postharvest loss [2]. Studying wound healing and identifying methods for shortening healing time is therefore important to reduce postharvest loss of potato tubers.

Various physical and chemical treatments have been shown to modulate wound healing in potato tubers [2]. For example, dipping tubers in hot water activated the phenylpropanoid metabolism and increased H_2_O_2_ content and peroxidase activity, promoting healing [4]. Abscisic acid accelerated the healing of tubers by enhancing the fatty acid and phenylpropanoid metabolism [5]. Salicylic acid and its analog, benzo-(1, 2, 3)-thiadiazole-7-carbothioic acids-methyl ester, promoted SPP and lignin accumulation by elevating phenylpropanoid metabolism [6]. *Candida oleophila*, which was originally isolated from the skin of apples, is a biocontrol yeast with properties of inhibiting postharvest diseases in fruit [7]. For example, *C. oleophila* was effective for controlling decay caused by *Penicillium digitatum* and *P. italicum* in citrus fruits [8], brown and soft rot caused by *Monilinia fructicola*, *Rhizopus stolonifer* in peaches [9], and anthracnose caused by *Colletotrichum gloeosporioides* in papayas [10]. Several companies (e.g., Aspire, BioNext, and Lesaffre International) have developed commercial *C. oleophila* products, which are used as biocontrol agents and are highly effective [7,11]. *C. oleophila* is a model biocontrol yeast and the only species whose mechanisms have been identified at the gene level [12]. Several modes of action, such as competition for nutrients and space, induction of pathogenesis-related genes in host tissues, and production of extracellular lytic enzymes, have been demonstrated to be a part of the biological control activity of *C. oleophila* through which it inhibits postharvest pathogens [13,14]. Among these, the prime modes of action are induced resistance and competition for nutrients and space [7]. Cai et al. [15] found that *C. oleophila* controlled against *P. expansum* in apples by inducing resistance and competing for space and nutrition. *C. oleophila* effectively inhibited *B. cinerea* by inducting pathogenesis-related protein gene expression in apples [14]. The yeast also induced systemic acquired resistance against *P. digitatum* by increasing phenylalanine ammonia lyase (PAL) activity and ethylene biosynthesis in grapefruits [13].

Although reports on the control of postharvest diseases of fruit by *C. oleophila* and its modes of action exist, no study has investigated its effect on the healing of potato tubers. The objective of this study was to determine the population dynamics of *C. oleophila* at the wound sites and weight loss and disease index during healing. We also aimed to observe and quantify the accumulation of SPP and lignin at wound sites, identify the key enzyme activities, study the gene expression of phenylpropanoid metabolism and its products, and measure the H_2_O_2_ content and peroxidase activity.

## 2. Materials and Methods

### 2.1. Tubers, Yeast, and Fungi

The potato tubers (*Solanum tuberosum* L. cv. Atlantic) were purchased from Ailan Potato Seed Co., Ltd., Gansu Province, China. Tubers without obvious defects or physical injuries and of appropriate size and appearance were selected. They were placed in mesh bags (7 kg per bag), brought to the laboratory within 4 h, and kept in darkness (20 °C ± 3 °C, RH 85–90%) before further use.

*C. oleophila* was purchased from the Chinese General Microbiological Culture Collection Center, Beijing (CICC 31860), China. The yeast was isolated from withered flowers by the Institute of Microbiology, Chinese Academy of Sciences. The yeast was maintained on nutrient yeast dextrose agar (NYDA) before further use.

*Fusarium sulphureum*, a major pathogen of potato dry rot, was provided by the Institute of Plant Protection, Gansu Agricultural Sciences Academy which was isolated from decayed potato tubers and cultured on potato dextrose agar medium (PDA) before further use.

### 2.2. Tuber Wounding

The wounding process was performed according to the process described by Lulai and Neubauer [3] with minor modifications. The tubers were first cleaned using tap water and then disinfected using sodium hypochlorite (0.1%, 3 min). Then they were finally rinsed with sterile deionized water. After air drying, the tubers were cut lengthwise in half using a sterilized knife (No. 2034, Deli, China).

### 2.3. C. oleophila Treatment and Wound Healing

The yeast was applied to tuber wounds sites as described by Zhang et al. [16] with minor modifications. First, *C. oleophila* was inoculated in 500 mL NYDA medium without added agar, that is, NYDB medium, and incubated on a shaker (28 °C at 220 rpm). The yeast cells were washed three times using sterile water, and the yeast concentration was then adjusted to 1 × 10^9^ CFU/mL with sterile water. Next, 100 μL *C. oleophila* or sterile water (control) were applied evenly to the wound using a sterilized applicator. All the tubers were air-dried, placed inside polyethylene bags with holes, and placed in the dark at room temperature (20 °C ± 3 °C, RH 85–90%) to heal.

### 2.4. Determination of C. oleophila Population Dynamics at Wound Sites

Population dynamics of *C. oleophila* were determined according to Cai et al. [15] with minor modifications. The entire wound was cut from the tubers, placed in sterile water (20 mL), and thoroughly ground using a mortar and pestle. The slurry was serially diluted five times; each dilution (100 μL) was evenly spread on the NYDA plates using a sterilized applicator. The number of colonies was counted after incubation for 48 h at 28 °C. The population dynamics of *C. oleophila* were expressed as log_10_ CFU/wound. Each individual wound served as one replicate, and the experiment was repeated three times at each sampling time.

### 2.5. Measurement of Weight Loss and Disease Index

The weight loss in wounded tubers was determined according to the gravimetric method at 0, 3, 5, 7, 14, 21, and 28 days after wounding; the results were expressed as percentages. Each treatment was replicated three times with nine tubers.

The disease index was determined as described by Jiang et al. [6]. Suspension of 1 × 10^6^ spores/mL of *F. sulphureum* was prepared, and 40 μL of the suspension was evenly coated on the wound surface using an applicator at different stages of healing. The tubers were placed in polyethylene bags with holes and the bags were stored. The disease index was evaluated 7 days after inoculation. The definition of no incidence level of a wound surface was 0, the surface incidence of 1/4 was 1, the surface incidence of 2/4 was 2, the surface incidence of 3/4 was 3, and the overall surface incidence was 4 grades. The disease index was calculated according to the following formula:

(1) $$\mathrm{Disease}\;\mathrm{index}=\frac{\Sigma \left(\mathrm{number}\;\mathrm{of}\;\mathrm{wounds}\times \mathrm{level}\;\mathrm{of}\;\mathrm{disease}\right)}{\mathrm{Total}\;\mathrm{wounds}\times \mathrm{highest}\;\mathrm{level}\;\mathrm{of}\;\mathrm{disease}}$$

### 2.6. Observation of SPP and Lignin Accumulation at Wounded Sites of Tubers

Slices were prepared according to the procedure by Jiang et al. [6]. The vertical cross-section of the wound was cut into thick slices (0.2–0.3 mm) using a double-edged razor blade. Autofluorescence of SPP was observed according to the procedure by Lulai et al. [17]. The cut sections were placed on glass slides and observed under a fluorescence microscope (BX53, Olympus, Tokyo, Japan) with an excitation filter of 450–490 nm. Lignin was observed according to the method described by Oirschot et al. [18], wherein the sections were stained with phloroglucinol (1% for 2 min) and hydrochloric acid (30 s) and then washed using deionized water. Lignin content was observed using an optical microscope (BX53, Olympus, Tokyo, Japan).

The thickness of SPP and lignin was determined using IS Capture software (Tucsen, Fujian, China).

### 2.7. Sampling

Sampling was conducted as described by Jiang et al. [6] with modifications. Healing tissues (3 mm deep including wounds) were obtained from the wound sites after 0, 1, 3, 5, 7, 14, 21, and 28 days. They were then grouped together, quick-frozen using liquid nitrogen, and ground into powder using a machine (IKA, Baden-Württemberg, Germany). Finally, the samples were individually packed in aluminum foil and stored at −80 °C.

### 2.8. Determination of Enzymes Activity

Phenylalanineammonialyase (PAL) activity was determined as described by Kozukue et al. [19]. A total of 3 g of sample powder was added to cold boric acid–borax buffer (5 mL, pH 8.8, 0.1 M), containing *β*-mercaptoethanol (5 mM·L^−1^), PVP (40 g·L^−1^), and EDTA (2 mM·L^−1^). Then it was centrifuged at 4 °C at 9000× *g* for 15 min. The reaction system included sodium borate buffer (3 mL, 0.05 M, pH 8.8), crude enzyme solution (0.5 mL, supernatant), and L-phenylalanine (0.5 mL, 20 mM). OD_0_ (0 h) and OD_1_ (1 h) at 290 nm were measured after placing the sample in a water bath (1 h, 37 °C); PAL activity was expressed as U·mg^−1^ prot.

Cinnamate-4-hydroxylase (C4H) activity was determined according to Lamb and Rubery [20]. For this, 3 g of sample powder was added in Tris-HCl buffer (2.5 mL, pH 8.9) containing crude enzyme (200 μL), G-6-PNa_2_ (6 μM), NADPNa_2_ (3 μM), and trans-cinnamic acid (8 μM). This reaction was terminated by adding HCl (6 M, 100 μL) after a water bath (25 °C, 30 min). The absorbance was determined at 340 nm; C4H activity was expressed as U·mg^−1^ prot.

4-coenzyme coenzyme A ligase (4CL) activity was determined according to Voo et al. [21]. A total of 3 g of sample powder was extracted using Tris-HCl buffer (3 mL, 150 mM, pH 8.0, glycerol (25%), and dithiothreitol (100 mM)). The reaction system (40 °C, 30 min) included: crude enzyme (0.5 mL), MgCl_2_ (0.5 mL, 15 μM), ATP (0.15 mL, 50 μM), coenzyme (0.15 mL, 1 μM), and *p*-coumaric acid (0.2 mL, 5 μM). Absorbance was determined at 333 nm. 4CL activity was expressed as U·mg^−1^ prot.

Cinnamyl alcohol dehydrogenase (CAD) activity was determined according to Goffner et al. [22]. A total of 3 g of sample powder was extracted using Tris-HCl buffer (5 mL, pH 7.5). The reaction system included: NADP (1 mL, 10 mM), crude enzyme (0.3 mL), and trans-cinnamic acid (1.2 mL). The reaction was terminated by adding HCl (100 μL, 6 M) after placing in a water bath (30 min, 37 °C). The amount causing a change of 0.001 in absorbance per min at 340 nm was defined as one unit (U). CAD activity was expressed as U·mg^−1^ prot.

Peroxidase (POD) activity was determined according to Venisse et al. [23]. A total of 3 g of sample powder was added in cold acetic acid sodium acetate buffer (5 mL, pH 5.5) containing PVPP (4%), PEG (1 mM·L^−1^), and Triton X-100 (1%). Then it was centrifuged (4 °C, 9000× *g*, 15 min). The reaction system included: guaiacol (3 mL, 20 mM) and crude enzyme solution (0.1 mL, supernatant). After H_2_O_2_ (0.2 mL, 0.5 mM) was added, the reaction began. The absorbance was determined at 470 nm. POD activity was expressed as U·mg^−1^ prot.

### 2.9. Determination of Protein Content

Protein content was determined according to Bradford [24]. The protein content was calculated by using bovine serum albumin as a standard protein and a standard curve.

### 2.10. Determination of SPP Monomer Contents

The contents of SPP monomers were determined according to Ayaz et al. [25]. The frozen powder (3 g) was immersed in acetone (30 mL, 70%), ultrasonically treated, and filtered. After filtration, redundant acetone was removed with nitrogen and extracted using ethyl acetate. The ethyl acetate was removed using a nitrogen stream (30 °C) and dissolved in methanol.

The Agilent 1101 with Eclipse Plus C18 column was used for high-performance liquid chromatography (HPLC) analysis. Acetonitrile (A) and acetic acid (1%) in water (B) constituted the solvent system. Gradient elution was performed as follows: 85% B, 0–5 min (1 mL min^−1^); 80% B, 5–7 min (1 mL min^−1^); subsequently, 70% B, (0.6 mL min^−1^, 25 °C). Under these conditions, samples (20 μL) were injected. The spectral information phenolic acid monomers were determined at 323 nm. The chromatograms of each standard were established. The phenolic acid contents were expressed as μg mg^−1^ prot.

### 2.11. Determination of Total Phenolic, Flavonoids, and Lignin Contents

The total contents of phenolic and flavonoids were determined according to Valcarcel et al. [26]. A total of 3 g of sample powder was added in cold acetic acid (10 mL, 0.5%) and acetone (70%); extraction occurred (20 °C, 24 h) in complete darkness. The extract was filtered and then centrifuged (4 °C 1000× *g*, 15 min). The mixed system included the extract (1 mL), Folin–Ciocalteu’s reagent (2 mL), and sodium carbonate solution (2 mL, 7.5%). The absorbance at 760 nm was then determined and expressed as mg GAE·100 g^−1^ FW. Each extract (3.5 mL) was mixed with aluminum chloride hexahydrate (0.25 mL, 10%) and sodium nitrite (0.25 mL, 5%); then, it was allowed to stand for 5 min and mixed with sodium hydroxide (1 mL, 1 mL^−1^). The absorbance of the mixture was read at 510 nm and expressed as mg CE·100 g^−1^ FW.

Lignin content was determined according to Morrison [27]. A total of 3 g of frozen tissue was added to cold ethanol (4 mL, 95%) and homogenized. After centrifuging at 4 °C at 9000× *g* for 15 min, the precipitate was separated using ethanol (95%) and ethanol:n-hexane (1:2 *v*/*v*) and washed three times. Then, the precipitate was dried in an oven (60 °C, 24 h) and dissolved in acetylacetonate acetic acid solution (1 mL, 25%). After placing in a water bath for 30 min (70 °C), NaOH (1 mL, 2 mL^−1^) to end the reaction was added. Glacial acetic acid (2 mL) and hydroxylamine hydrochloride were added (0.1 mL 7.5 mL^−1^). After centrifuging at 4 °C at 12,000× *g* for 30 min, the absorbance values at 280 nm of the filtrate were determined; lignin content was expressed as unit A280·g^−1^ FW.

### 2.12. Total RNA Extraction and qRT-PCR Analysis

qRT-PCR analysis was performed according to Woolfson et al. [5] with minor modifications. Total RNA was isolated from 3.0 g of frozen tissue using RNAsimple Total RNA Kit (Cat. No. DP419, TIANGEN Biotech, Beijing, China). This isolated total RNA was used to generate cDNA using FastKing RT Kit (Cat. No. KR116, TIANGEN Biotech, Beijing, China), and qRT-PCR was performed using a Roche Light Cycler 480 (Roche, Basel, Switzerland) following the manufacturer’s protocols. The 2^−ΔΔCt^ method was used for relative quantification. The primers and *ef1* (Supplementary Materials Table S1) were designed according to Woolfson et al. [5] and Nicot et al. [28].

### 2.13. Determination of H_2_O_2_ Content

The quantification of H_2_O_2_ was performed as described by Bajji et al. [29] with slight modifications. A total of 3 g of sample powder was mixed with 2 mL of a reagent mixture (pH 7.0, potassium phosphate (50 mM), guaiacol (0.05%), and horseradish peroxidase (10 units mL^−1^)). After incubation in the dark for 2 h at 20 °C, centrifugation was performed at 9000× *g* for 15 min at 20 °C. The absorbance of the supernatant was measured at 470 nm, and H_2_O_2_ content was expressed as μM·g^−1^ FW.

### 2.14. Statistical Analysis

The aforementioned experiments were repeated in triplicate at each time point. The average and standard errors (±SE) of data were determined using Microsoft Excel 2010. Significant differences were analyzed using analysis of variance (SPSS 17.0 Inc., Chicago, IL, USA), followed by Duncan’s multiple range tests (*p* < 0.05).

## 3. Results

### 3.1. Population Dynamics of C. oleophila at Wounded Sites

The growth dynamics of *C. oleophila* at wounds reflect its ability to compete against pathogens during healing in terms of nutrition and space. *C. oleophila* exhibited rapid proliferation at Day 3 of healing and slow growth during Days 3-7. The number of yeast cells reached the maximum value of 2.2 × 10^11^ CFU mL^−1^ on Day 7, which was 20.3 times higher than that on Day 0. After Day 7, the number of yeast cells began to decrease rapidly. The quantity was 5.3 × 10^7^ CFU mL^−1^ on Day 28, which was 1/18 of the quantity of Day 0 (Supplementary Materials Figure S1). These results indicate that *C. oleophila* can colonize wound sites and multiply quickly on the surface of the wound during healing

### 3.2. C. oleophila Reduced Weight Loss and Disease Index

**Figure 1:**
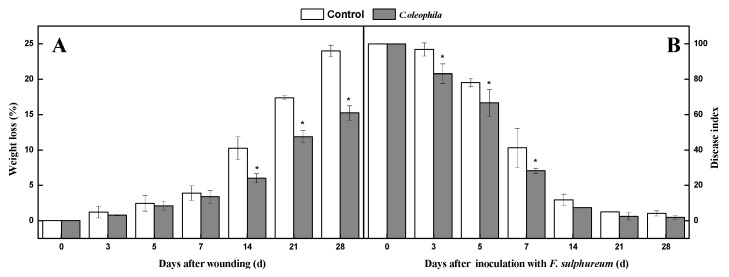
Effect of *C. oleophila* treatment on weight loss (**A**) and disease index (**B**) of tuber during healing. Bars indicate standard error (±SE). Asterisks indicate significant difference (*p* < 0.05).

Weight loss and disease index are crucial factors for estimating the ability of healing. During healing, weight loss in all wounded tubers increased continuously. The weight loss of treated tubers was significantly lower; which was 71.4% lower than the control after 14 days (*p* < 0.05) (Figure 1A). During healing, the disease index in all tubers decreased continuously. The disease index of the treatment was reduced by *C. oleophila* to a value of 45.6% lower than that of the control after 7 days (*p* < 0.05) (Figure 1B). The weight loss and disease index indicated that *C. oleophila* promoted the healing of wounded tubers.

### 3.3. C. oleophila Accelerated Accumulation of SPP and Lignin

**Figure 2:**
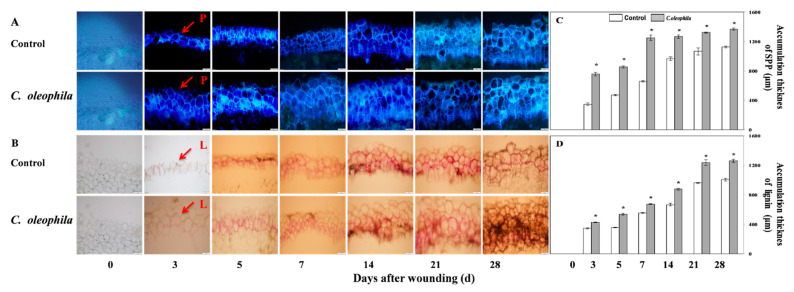
Effect of *C. oleophila* treatment on the accumulation of SPP (**A**) and lignin (**B**) and cell layers thickness of SPP (**C**) and lignin (**D**) at wounded sites of tubers during healing. Red arrows indicate SPP and lignin deposited in cell walls and are marked as “P” and “L”, respectively. Bars indicate standard error (±SE). Asterisks indicate significant difference (*p* < 0.05).

The SPP and lignin depositions reflect the speed and ability of healing in tubers. The SPP accumulation increased continuously during healing in all tubers. The SPP accumulation was broader and faster in the treated tubers than in the control (Figure 2A). The thickness of the cell layers of SPP accumulation in all tubers also continuously increased. However, the treatments exhibited a significantly thicker layer than the control; the thickness in the treatment was 90.0% higher than that in the control after 5 days (*p* < 0.05) (Figure 2C). Similarly, lignin accumulation of all tubers increased continuously during healing, and the accumulation in the treatment was significantly faster than that in the control (Figure 2B). The thickness of cell layers of lignin increased in all tubers; that of treated tubers was 26.1% higher than that of the control after 21 days (*p* < 0.05) (Figure 2D). The accumulation of SPP and lignin indicated that *C. oleophila* accelerated SPP and lignin deposition at the wound sites.

### 3.4. C. oleophila Activated Key Enzyme of Phenylpropanoid Metabolism

**Figure 3:**
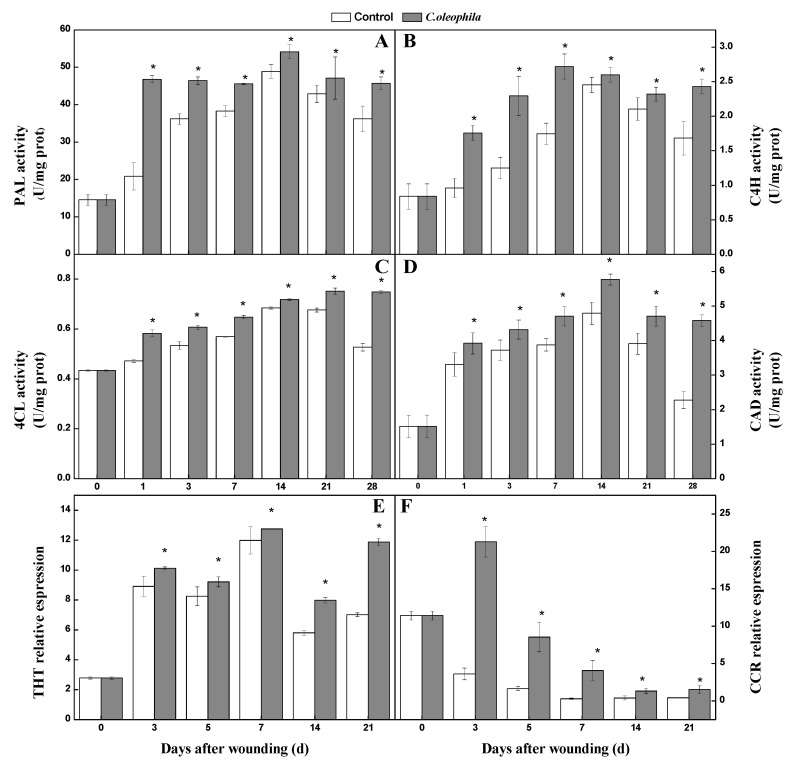
Effects of *C. oleophila* treatment on the activity of PAL (**A**), C4H (**B**), 4CL (**C**), and CAD (**D**) and *THT* (**E**) and *CCR* (**F**) expression at wound sites of tubers during healing. Bars indicate standard error (±SE). Asterisks indicate significant difference (*p* < 0.05).

The activity of the key enzymes of the phenylpropanoid metabolism reflects the ability of phenolic production. During healing, PAL activity in treated tubers showed a rapid increase after 3 days and then became stable. However, control tubers exhibited a continuous enhancement during healing and peaked at Day 14. The PAL activity in the treatments was significantly (1.24 times) higher than that in the controls on Day 3 (*p* < 0.05) (Figure 3A). Also, C4H activity in all tubers first increased and then decreased. The activity in the treatments was 83.5% higher than that in the control at Day 7 (*p* < 0.05) (Figure 3B). 4CL activity in the treatments increased slowly and then remained stable. The activity in the controls increased slowly and then decreased. 4CL activity in the treatments was 42% higher than that in the control after 28 days (*p* < 0.05) (Figure 3C). The CAD activity of all tubers exhibited a gradual increase and then a decrease. The CAD activity of treatment was 1.01 times higher than that of the control after 28 days (*p* < 0.05) (Figure 3D). During healing, *THT* gene expression in the treatment was higher than that in the control. After 21 days, gene expression of the treatment was 61.9% higher than that of the control (*p* < 0.05) (Figure 3E). *CCR* gene expression in the treatment was 4.9 times higher than that in the control after 1 day (*p* < 0.05) (Figure 3F). These results demonstrate that *C. oleophila* significantly enhanced the key enzyme activities of phenylpropanoid metabolism, and elevated *THT* and *CCR* gene expression.

### 3.5. C. oleophila Enhanced the Content of Phenolics and Lignin

**Figure 4:**
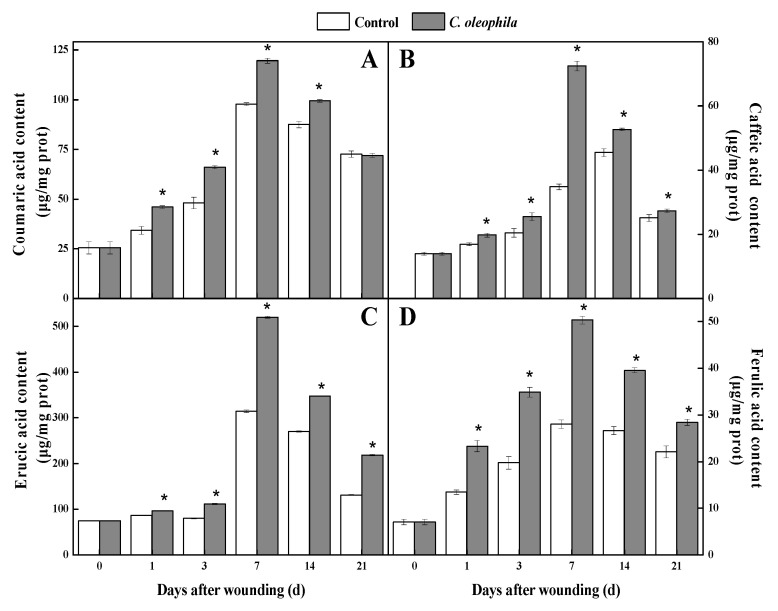
Effects of *C. oleophila* treatment on the content of coumaric acid (**A**), caffeic acid (**B**), erucic acid (**C**), and ferulic acid (**D**) at wound sites of tubers during healing. Bars indicate standard error (±SE). Asterisks indicate significant difference (*p* < 0.05).

**Figure 5:**
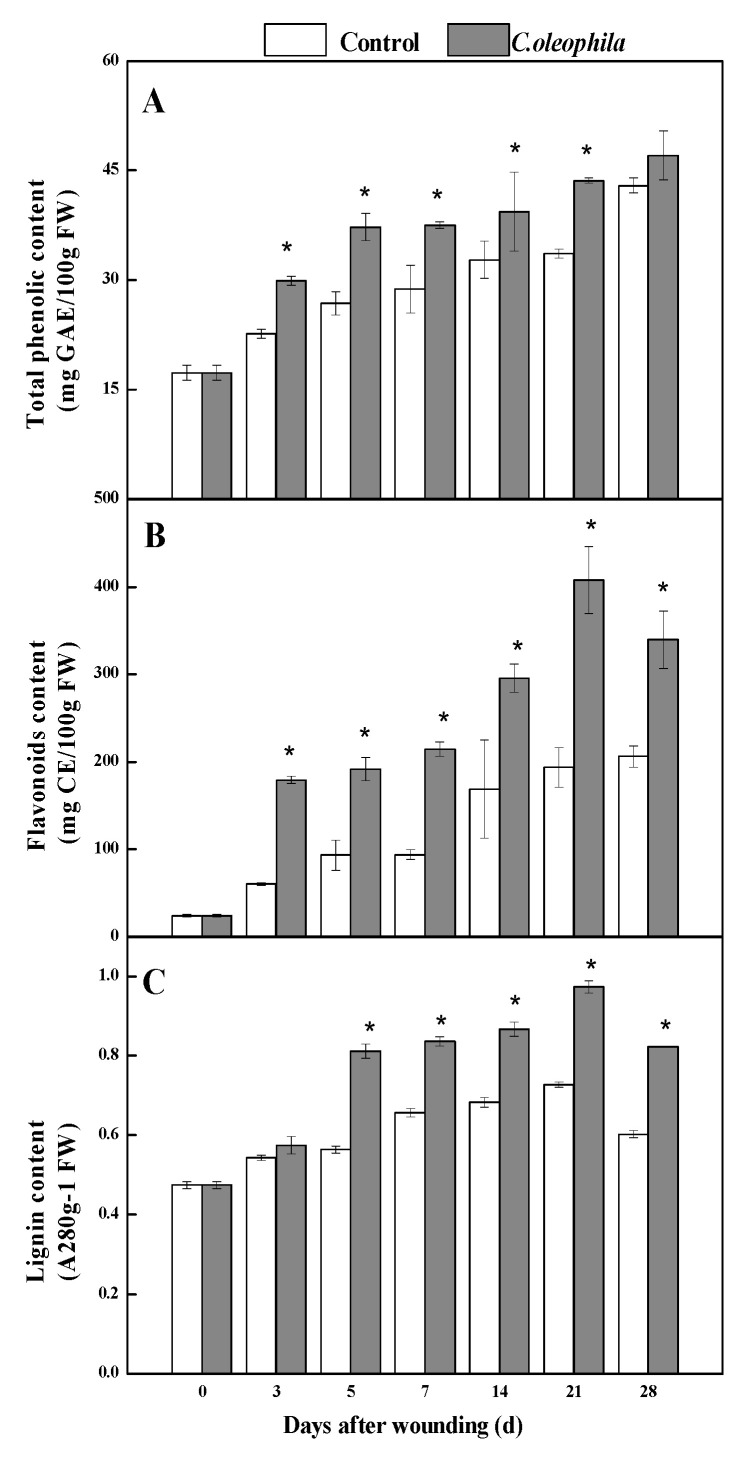
Effects of *C. oleophila* treatment on the content of total phenolic (**A**), flavonoids (**B**), and lignin (**C**) at wound sites of tubers during healing. Bars indicate standard error (±SE). Asterisks indicate significant difference (*p* < 0.05).

Phenolics are the most critical substrates of SPP and lignin. During healing, the content of four phenolic acids initially increased and then decreased in the treatments and controls. The coumaric acid content of the treatments was substantially higher (37.5%) than that of the controls after 3 days (*p* < 0.05) (Figure 4A). Caffeic acid and erucic acid content in the treatments were clearly higher (1.1 times and 65.3%, respectively) than those in the control after 7 days (*p* < 0.05) (Figure 4B,C). The ferulic acid content of the treatments was substantially higher (79.6%) than that of the control, at Day 7 (*p* < 0.05) (Figure 4D). The total phenolic continuously increased in all tubers, and was significantly higher (39.0%) in the treatment than it was in the control on Day 5 (*p* < 0.05) (Figure 5A). The flavonoid content first increased and then decreased in all tubers. The flavonoid content of the treatment was 1.8 and 1.3 times higher than that of the control at Days 3 and 7, respectively (*p* < 0.05) (Figure 5B). The lignin content of the treatment was significantly higher (43.8%) than that of the control after 5 days (*p* < 0.05) (Figure 5C). The results show that the yeast increased the content of phenolic acids, flavonoid, and lignin at the wound sites.

### 3.6. C. oleophila Increased the H_2_O_2_ Content and POD Activity

**Figure 6:**
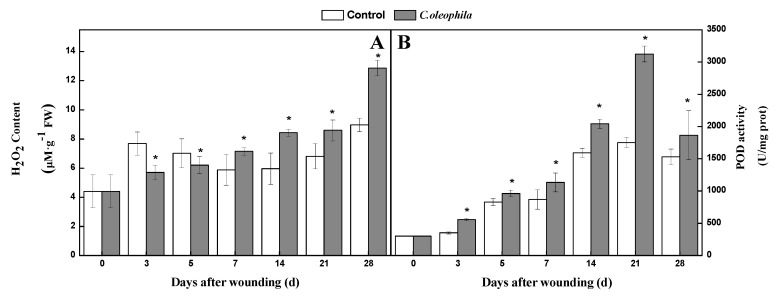
Effects of *C. oleophila* treatment on the content of H_2_O_2_ (**A**) and the activity of POD (**B**) at wound sites of tubers during healing. Bars indicate standard error (±SE). Asterisks indicate significant difference (*p* < 0.05).

H_2_O_2_ and POD play an essential role in healing by facilitating oxidative crosslinking of SPP and lignin substrates. During healing, the H_2_O_2_ content of treated tubers increased. However, the control first increased, then decreased, and finally increased again. The H_2_O_2_ content in the treatment was substantially lower than that in the control before Day 7. However, it was significantly higher than that in the controls after Day 7. The H_2_O_2_ content in treated tubers was 43.7% higher than that in the control after 28 days (*p* < 0.05) (Figure 6A). During healing, the POD activity of all tubers first increased and then decreased. POD activity in the treated tubers was 78.5% higher than that in the control at Day 21 (*p* < 0.05) (Figure 6B). The results demonstrate that the yeast increased H_2_O_2_ content and POD activity at the wound sites.

## 4. Discussion

In this study, *C. oleophila* was found to proliferate rapidly at the wound sites of tubers during the early and intermediate healing stages. This proliferation is similar to the growth dynamics of *C. oleophila* at wound sites of apples [30]. These wounds resulted in cell rupture and exudation of nutrients, providing a sufficient basis for yeast proliferation [15]. Because of the quick colonization and proliferation of yeast cells in the wound sites, the available nutrients are consumed and the space is occupied by *C. oleophila* [14]. This, in turn, leads to a lack of nutrients and living space for inoculated pathogens [15]. Therefore, the infection with inoculated pathogenic fungi is inhibited. With the incrassation of SPP and lignin at wound sites during intermediate and later stages, nutrition from within the wound is blocked. A large number of yeast cells die, forming a protective layer that inhibits evaporation [16].

Phenylpropanoid metabolism plays a vital part in the healing of tubers, providing precursor substances for SPP and lignin and producing antifungal and antioxidant polyphenol [2,3]. PAL, a pivotal enzyme of phenylpropanoid metabolism, catalyzes the conversion of L-phenylalanine to trans-cinnamic acid which is the first committed step in the pathway [1]. C4H catalyzes cinnamic acid change to *p*-coumaric acid [6]. Then, 4CL catalyzes the conversion of *p*-coumaric acid and sinapic acid to hydroxycinnamoyl-CoA, a central intermediate for numerous phenylpropanoid metabolites [1]. Two main catalytic pathways exist, hydroxycinnamoyl-CoA is a substrate of THT, which links hydroxycinnamic acids with tyramine and catalyzes the formation of SPP monomer [5]. Also, CCR and CAD catalyze the hydroxycinnamoyl-CoA to precursor substances of three lignin monomers [31]. The flavonoids, as producers of phenylpropanoid metabolism, have stronger antioxidant and antifungal abilities [6]. Flavonoids can directly kill the fungal pathogen, enhancing disease resistance [32]. Scervino et al. [33] found that the germination of spores of *Gigaspora margarita* was inhibited by most of the flavonoids. Other research showed that flavonoids inhibited the hyphal growth of *Gigaspora* and *Glomus* [34]. In this study, *C. oleophila* increased the activity of key enzymes and the production of phenylpropanoid metabolism, which is comparable with the observation of Cai et al. [15] who found that *C. oleophila* induced defense response in apples by most flavonoids. The components *β*-glucan, chitin, and mannan are the main structural constituents of yeast cell walls [35]. A previous study showed that resistance of pears against *P. expansum* was induced by (1–3)-*β*-D-glucan through improving defense-related gene expression and PAL activity [36]. Also, chitin increased the activities of defense-related enzymes and gene expression, inducing the resistance of tomatoes against *B. cinerea* [35]. Thus, we infer that the *β*-glucan or chitin of the yeast cell wall could activate phenylpropanoid metabolism, increasing the accumulation of phenolic substrates at wound sites of tubers.

As the most critical components of wound tissue, SPP and lignin provide primary protection for wounds [1]. Coumaric acid, caffeic acid, erucic acid, and ferulic acid are monomers of SPP. These monomeric substances are synthesized in the cytoplasm, transported to the cell, and covalently connected by peroxidase and H_2_O_2_ [2]. SPP provides durable and strong protection against resistant infection and evaporation of wounds in tubers [1]. Lignin is composed of sinapisol, coniferol, and *p*-coumarin and polymerized through the action of peroxidase and H_2_O_2_. Lignin, as a component of the secondary wall of the cell wall [31], constitutes a physical barrier for the wound [2]. On the one hand, lignin increases the mechanical strength of the organization, increasing resistance ability; on the other hand, as a mechanical barrier, it restricts pathogens from obtaining nutrients from the surrounding host tissue, preventing the spread of fungi to the surrounding tissue [37].

SPP and lignin polymerization involve the oxidative crosslinking of H_2_O_2_ and POD [2]. During healing, H_2_O_2_ primarily derives from superoxide anions via NADPH oxidase (NOX) pathway [38]. Wounds exhibit an influx of Ca^2+^ into the cells of potato tubers. Ca^2+^ combines Ca^2+^ dependent protein kinases, inducing the phosphorylation of respiratory burst oxidase homolog and NOX transfers electron to O_2_, producing O_2_^−^ [39]. During the healing process of potato tubers, O_2_^−^ rapidly decomposes into H_2_O_2_ under the action of superoxide dismutase [29]. In addition, polyamine oxidase and cell-wall-bound POD have been shown to be crucial sources of H_2_O_2_ formation [40]. In this study, *C. oleophila* significantly improved H_2_O_2_ content. This is consistent with the results reported for apple wounds and *Pichia. guilliermondii* treatment [16]. The research showed that chitin increased intracellular calcium levels, leading to the activation of *Arabidopsis* NOX [41]. In addition, chitin treatment promoted the accumulation of ROS by inducing gene expression and activity of SOD in tomatoes [35]. Thus, we infer that chitin from the cell walls of yeast increased intracellular calcium levels, NOX, and SOD activities, producing more H_2_O_2_. However, this process requires further study. POD is involved in the oxidative crosslinking of SPP and lignin monomers, of which the expression was rapidly up-regulated after wounding in tubers [3]. In this study, we found that *C. oleophila* enhanced POD activity, which is similar to the findings of [13] concerning grapefruit. Research has shown that chitin induced POD activity and its corresponding gene expression in tomatoes [35]. Therefore, we assume that chitin from cell walls enhances POD activity.

## 5. Conclusions

*C. oleophila* can colonize the tuber wound sites and quickly proliferate. In this study, it was found to reduce the disease index of inoculated tubers through competition for nutrition and space and reduced weight loss by forming a protective layer of dead cells. *C. oleophila* activated the key enzyme for phenylpropanoid metabolism, increased the content of ferulic, caffeic, coumaric, and sinapic acids, as well as that of total phenolic, flavonoid, and lignin at the wound sites. The yeast also enhanced H_2_O_2_ content and POD activity, which promoted oxidative crosslinking of phenolics, thus forming SPP and lignin. The deposition of SPP and lignin at wound sites resulted in the reduction of weight loss of wounded tubers and the reduction of the disease index.

## Data Availability

No new data were created or analyzed in this study. Data sharing is not applicable to this article.

## Supplementary Materials

The following are available online at https://www.mdpi.com/article/10.3390/foods10061286/s1, Figure S1: Population dynamics of *C. oleophila* on wounds of potato tuber during healing. Bars indicate the standard errors (±SE), Table S1: Gene primer sequence of genes related to the synthesis of suberin and lignin.
