# Supplementary material for: Candida Oleophila Proliferated and Accelerated Accumulation of Suberin Poly Phenolic and Lignin at Wound Sites of Potato Tubers
Source: Foods. 2021 Jun 4;10(6):1286. doi: 10.3390/foods10061286 (PMC8230253; doi:10.3390/foods10061286)
Supplement: Supplementary file 1 [file foods-10-01286-s001.zip › foods-1198914-supplementary.pdf]

Supplementary Materials

Supplementary Fig. S1. Population dynamics of *C. oleophila* on wounds of potato tuber during healing. Bars indicate the standard errors (±SE). Different letters indicate significant difference ($P < 0.05$).

Supplementary Table. S1. Gene primer sequence of genes related to the synthesis of suberin and lignin.

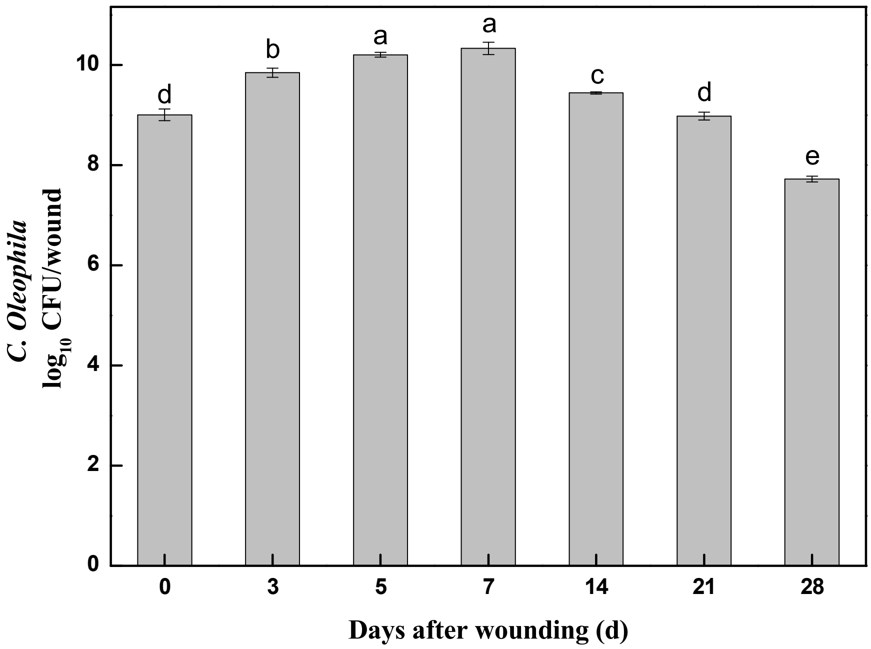

Supplementary Fig. S1.

Genes	NCBI Accession No.	Primer sequence (5’–3’)
StTHT	AB061243.1	Forward: AGGTATGGCAAATTGCATGGTG
		Reverse: TGTCTCTTCCTCAATTTTCCCCT
StCCR	AY149608.1	Forward: GAGCCAGCGGTTATAGGGAC
		Reverse: TCCACAACTTTATCCGGGGC
Efla*		Forward: CAAGGATGACCCAGCCAAG
		Reverse: TTCCTTACCTGAACGCCTGT

* From Nicot and Evers (2005).

Supplementary Table. S1.
